# Spectrum of intracranial tumours in a tertiary health carefacility: our findings

**DOI:** 10.11604/pamj.2015.20.24.4935

**Published:** 2015-01-09

**Authors:** Sunday Sokunle Soyemi, Olugbenga Olayide Oyewole

**Affiliations:** 1 Department of Pathology and Forensic Medicine, Lagos State University TeachingHospital, Ikeja, Lagos, Nigeria; 2 Department of Oncology, Lagos State University Teaching Hospital, Ikeja, Lagos, Nigeria

**Keywords:** Magnetic resonance imaging, intracranial, WHO

## Abstract

**Introduction:**

Primary brain tumours are uncommon with an annual incidence of 5-10/100000. This study has attempted to analyse the histological pattern of intracranial tumours seen in our centre.

**Methods:**

A retrospective study of cases of intracranial tumours seen was conducted over a period of 5 years ie from January 2008 to December 2012. All the slides were reviewed. The age, sex, diagnosis using the WHO grading and the histological subtypes were recorded. Data were analysed using the (SPSS) Software version 17.

**Results:**

Altogether, 56 cases of intracranial tumours were seen out of a total of 12,610biopsies representing 0.004%. The male to female ratio (M: F) was approximately 1:1.1The mean age of the patients was 36 ± 20.35 (range, 2 to 85). Astrocytomas accounted for 30% (17)while 29% (16) had Meningioma. Medulloblastoma accounted for 18%.(10). Of the cases of Gliomas, majority(52%) fell under WHO grade II. (38%)of the Meningioma were of the mixed type while 25% had transitional type.

**Conclusion:**

Astrocytomas was the commonest brain tumour. These patterns corroboratedmost studies that have been done. Metastasis to the brain was however, not seen in this study.

## Introduction

During embryogenesis the neuroectoderm differentiates into the neuroepithelium from which three primitive tissue are derived namely; the neuroblast, thespongioblast and the primitive ependymal cells. Intracranial neoplasms may arrive at any level of differentiation of these cells [1]. Brain tumours are a diverse group of primary CNS tumours and secondary neoplasms arising either from the brain or from haematogenous spread from distant sites. Each tumour has a distinctivebiology, treatment and prognosis [2].

The annual, global, age standardized incidence of primary malignant intracranial tumours is approximately 3.7 per 100,000 for males and 2.6 per 100,000 for females [3, 4]. Gliomas are common tumours in adults and paediatric age group. In the adult population, Anaplastic Astrocytoma and Glioblastoma Multiforme are the most common glial tumours with an annual incidence of 3 to 4 per 100,000 populations [5]. Although few studies had been done on the epidemiology of intracranial tumours in Nigeria and Africa as a whole, the objective of this paper is to provide an AMPLE and detailed perspective on the epidemiological studies of intracranial tumour in a newly growing tertiary health facility in Lagos, Nigeria.

## Methods

A retrospective study of cases of intracranial tumours seen was conducted over a period of 5 years ie from January 2008 to December 2012. These were Nigerian patients who had surgery and their samples were sent to our histopathology department for diagnosis. The total sample seen over this period was relatively small when compared to other studies. This was due to the fact that the neurosurgery department was new and had only one neurosurgeon. The specimens were processed in paraffin and stained with eosin and haematoxylin. In rare cases special stains were used to demonstrate neuroglial cells, reticulin and collagen fibres. All the slides were reviewed. The age, sex, diagnosis using the WHO grading and the histological subtypes were recorded. Data were analysed using the (SPSS) Software version 17.

## Results

Altogether, 56 cases of intracranial tumours were seen out of a total of 12,610 biopsies representing 0.004% of the surgical biopsies. The male to female ratio (M: F) was approximately 1:1.1. The mean age of the patients was 36 ± 20.35 (range, 2 to 85). Astrocytomas accounted for 30% (17), 29% (16) had Meningioma while Medulloblastoma accounted for 18%. Pituitary adenoma and Ependymomas represented 14% and 9% respectively (See Table 1). Of the cases of Astrocytic tumours, (Table 2) majority (52.9%) fell under WHO grade II this is followed by Glioblastoma Multiforme which represented 35.2%. In Table 3, 37.5% of the Meningiomas were of the mixed type while 25% had transitional type. Meningothelial, Fibroblastic and Psammomatous each had 12.5%.

**Table 1:** Frequency and percentage distribution of the different types of tumour

Tumour	Frequency	Percentage
Astrocytomas	17	30%
Meningioma	16	29%
Medulloblastoma	10	18%
Pituitary adenoma	8	14%
Ependymoma	5	9%
Total	56	100%

**Table 2:** Percentage distribution of the different grades of Astrocytoma

Astrocytomas	Percentage
Grade 1	5.9%
Grade 2	52.9%
Grade 3	5.9%
Grade 4	35.2%
	100%

**Table 3:** Frequency and percentage distribution of the different types of meningioma seen I this study

Type of Meningioma	Frequency	Percentage
Mixed	6	37.5%
Transitional	4	25%
Meningothelial	2	12.5%
Fibroblastic	2	12.5%
Psammomatous	2	12.5%
Total	16	100%

## Discussion

**Figure 1:**
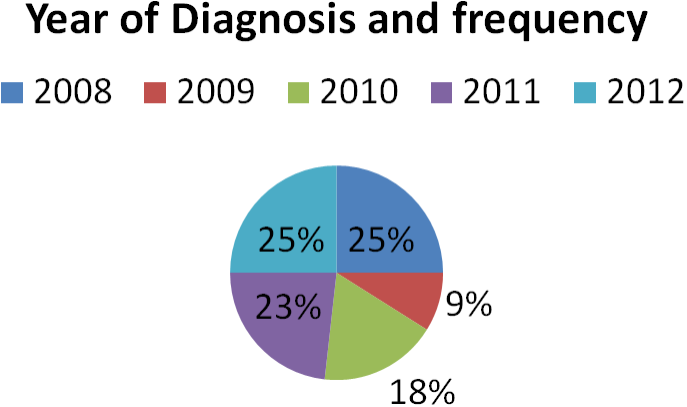
Distribution of the frequency in each year from 2008 to 2012

**Figure 2:**
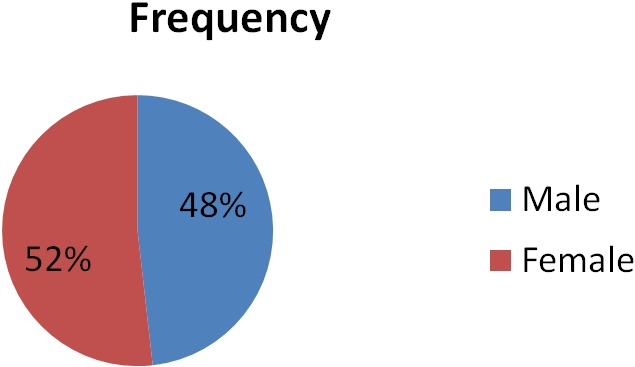
Male to female distribution of the intracranial tumours

In this study, the lowest cases were seen in year 2009 while the highest were seen in 2008 and 2012 (Figure 1). The male to female ratio was 1:1.1 with no significant gender difference (Figure 2). Other studies have reported insignificant gender difference [1, 2]. The peak age of occurrence in adult was 21-30 years with only seven cases seen in children. Only one case of intracranial tumour was seen in person above 70 years. This is closely compatible to a similar study done over an eleven year period in Ibadan, South West Nigeria [6] ie from 1980 to 1990 which revealed a ratio of 1:1. However a twist is seen in the high occurrence of metastatic tumours which came next to Astrocytoma in the same study done by Olasode in Ibadan, Nigeria.

In our study, no single case of metastasis to the brain was found. The reason may likely be due to the relatively small numbers (56) compared to the study in Ibadan (210) of eleven years duration. Early detection of cancers by newer diagnostic methods and sophisticated treatment options in this decade compared to the last two decades might also be a contributing factor for the absence of intracranial metastasis. In this study Astrocytoma was the most common intracranial tumour. Other studies have also revealed the predominant nature of Astrocytoma [7–10].

Meningiomas have been found to be high in Africans and constitute approximately 20% of intracranial tumours [10]. In our study, it accounted for 29%. This figure is relatively higher than other previous works [9–11] although closely compatible with a similar study in Iran [12] and in Egypt [13]. It is worthy of note that an unequivocal factor that has been implicated in Meningioma is irradiation of the cranium even at low doses [14, 15].

Some other studies which have tried to establish a relationship between the development of brain neoplasm and head injuries, exposure to high tension wires, use of cellular phones and dietary exposure to N-nitrosoureas are really not convincing and are also controversial [16–19]. The changing trend in meningioma and it's associated hypothesis would presently serve as an area of future research that can be prioritized.

One of the most common tumours of the posterior fossa is Medulloblastomas which is highly invasive. This tumour also has a high tendency to recur and spread through the CSF space, subsequently making radical cure of the tumour a problem [2]. In this study, it accounted for 18%. This is compatible with most Caucasian studies where itaccounts for 12-25% of all paediatric CNS tumours [20].

Pituitary adenoma constituted 14% of all tumours. This is compatible with similar studies done in Ghana [11] Egypt [13] and Nigeria [6, 9, 11].

## Conclusion

In conclusion, astrocytoma and meningioma are the most common intracranial tumours in our study.
